# Which Health Systems Provide the Most Health?

**DOI:** 10.1089/pop.2019.0218

**Published:** 2020-11-26

**Authors:** R. Scott Braithwaite

**Affiliations:** Department of Population Health, New York University School of Medicine, New York, New York, USA.

**Keywords:** health system performance, Health Value Unit, health-adjusted life-years

Imagine choosing a health system based on the health of those members who are most similar to you (Fig. 1). Consider how health markets would transform if health system reimbursement were based primarily on the amount of health added by each system. Envision employers choosing health systems knowing which ones best preserved employees' productivity. Although these scenarios were formerly imaginary, advances in technology and prediction now make them possible.

**FIG. 1.**
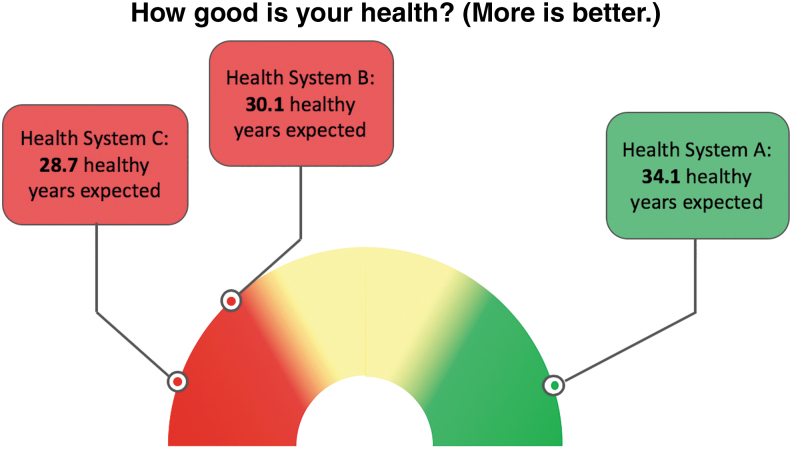
Hypothetical comparison of health and well-being of “people like you” simulated in Health Systems A, B, and C. “People like you” are those of the same age, sex, education, income, and racial/ethnic group; and who have the same health conditions and health risks that you do right now. For this hypothetical example, “you” are a 42-year-old, female, lower-income, rural, white, and obese smoker with prediabetes and stage 2 hypertension. Compared to Health Systems B and C, people like you in Health System A live longer because of better preventive care that reduces the risk of common diseases (eg, cardiovascular disease); however people like you in Health System A have more unexpected deaths (eg, suicide) and lower well-being than people like you in Health Systems B and C.

## Measuring Health Is Now Possible

The amount of health added by a health system is no longer inestimable. Advances in computing power and predictive analytics enable accurate group-level estimations with predictors that are mostly available in existing administrative and clinical databases. For example, our group estimated the amount of health added by alternative investment decisions to guide goal setting in a large integrated health system.^1^ Estimations can be performed based on mechanistic models,^2,3^ statistical models,^4^ machine learning, or hybrid models that attempt to disentangle prediction from causation.

What would estimating health added look like? Assume measurements employ health-adjusted life-years (HALYs), such as quality-adjusted life-years or disability-adjusted life-years, meaning the “adjustment” is guided by preferentially equivalent changes in length of life.^5^ Imagine a woman turning 42 years old begins that year with a health-adjusted life expectancy of 35.0 HALYs, and ends that year with 34.8 HALYs, a decline of 0.2 HALYs. Because an average 42-year-old woman loses 0.9 HALYs over 1 year, her health system has facilitated a gain of 0.7 HALYs (0.2 HALYs minus −0.9 HALYs). If reimbursement were linked to health added, assuming 0.01 HALYs per Health Value Unit (HVU), her health system would be credited 70 HVUs for her care. Alternatively, imagine a 55-year-old woman with stage 3 breast cancer who is cured, increasing her health-adjusted life expectancy from 5.0 HALYs to 35 HALYs. Her health system would be credited 3000 HVUs. Although estimating HVUs may seem complicated, it is arguably easier than estimating resource-based relative value units, the current unit often indexed in reimbursement decisions, yet would be far more efficient.

A possible objection to the principle of paying for health is that if stage 3 breast cancer remained uncured despite best possible care, no reimbursement would occur. But when averaged across in-care populations and suitably risk adjusted, the principle of paying for health would be fair and would incentivize health. These kinds of impacts (eg, HALYs gained/restored) are happening quite often, in every practice and in every system. But although some are doing more of it than others, we do not measure this important impact.

## Other Advantages of Reimbursing Quantity of Health Added

Reimbursing quantity of health added would bring other advantages besides aligning financial incentives with health improvement. Health systems would pursue “health added” analogously to how businesses pursue “value added,” increasing efficiency. Personalized medicine would be incentivized because assessing individualized risks and benefits helps maximize the quantity of health added. Patient-centered care would be incentivized because considering individualized preferences is necessary to maximize adherence with healthful therapies and behaviors. Bias favoring treatment over prevention would decrease because reimbursement would not change based on whether health is added through treatment or prevention. Clinician burnout and micromanagement may decrease because one intuitive metric would eclipse a multitude of rapidly changing metrics with questionable relevance to the health of particular patients.

## Some Reasons Why We Do Not Know Which Health Systems Provide the Most Health

Reasons why we do not know how much health is added by health systems include priorities of health system leadership, inertia of regulatory authorities, and consumers not viewing health systems as sources of wellness.

### Priorities of health system leadership

Leadership often prioritizes aspects of health system performance with bottom line impact, particularly reimbursement and market share. Adding health does not necessarily increase reimbursement or improve market share, aside from unusual circumstances when quality-based performance bonuses align closely with quantity of added health. Assessing health added might even harm the bottom line if results are unfavorable. Additionally, highlighting the amount of health added is untested as a marketing strategy.

### Regulatory inertia

Regulation and quality assessment of health care has become a lucrative industry. Entities invested in the status quo oppose changes that would increase costs without necessarily increasing revenue, such as changes in measurement tools. There is inertia once business models and industries develop around particular measures and outcomes, even if these measures and outcomes do not capture what is most important.

### Consumer demand

People with active health problems may choose health systems based on access to a particular provider, institution, or patient experience; or on cost rather than on amount of added health. People without active health problems may view health system performance as a contingency (eg, what if I became sick?) rather than a certainty, a different vantage point from other wellness-oriented industries where maintaining or improving health is paramount.

## Policy Implications

Two pathways could elevate HVUs as a health system performance metric. First, the Centers for Medicare & Medicaid Services could link reimbursement to quantity of health added, phased in concurrently with existing payment models such as Accountable Care Organizations. Other payers likely would follow, as has occurred historically. Second, a disruptive innovation could occur if health systems can gain market share through horizontal integration, co-marketing or co-branding with wellness providers (eg, diet organizations, fitness centers, spas). Health systems might market membership as a wellness product for otherwise healthy people rather than a contingency in case of illness. Conversely, wellness providers may find it advantageous to offer scientifically validated health improvement in addition to targeting conditions such as weight loss and mindfulness.

### Limitations

Although paying for health has unrealized potential to align health system activities with health improvement, it is not a panacea. It is more suited to chronic disease and symptom management than to acute disease flare-ups or injuries. Accordingly, reimbursements for health added should be supplemented by reimbursements for a parsimonious number of additional health care domains such as patient satisfaction in the “Triple Aim.”^6^ Model-based health projections will always be inaccurate sometimes, particularly when a person has an impactful disease that is not considered by the predictive model. However, this concern can be ameliorated by specifying when the model is likely to be inaccurate and therefore should not apply.

Another concern is that incentivizing health might lead health systems to preferentially enroll healthier persons. However, this concern is unjustified. Unhealthier people have greater potential health gains than healthier people, so reimbursement is potentially greater. Reimbursement can be risk stratified based on social risks, with the caveat that too much risk stratification may perpetuate health disparities.^7^

Reimbursing health added could potentially be ageist, because older persons have fewer healthy life-years to gain. However, ageist bias can be remedied by scaling the health benefit based on age-specific health expectancies. For example, adding 0.5 HALYs to 5 HALYs (ie, 85-year-old) could be valued equivalently to adding 3 HALYS to 30 HALYs (ie, 50-year-old).

A more intractable concern is that reimbursing added health could incentivize health systems to hide or postpone diagnoses that reduce health-adjusted life expectancy. However, all reimbursement systems are gameable. This concern could be proactively anticipated and addressed by anti-fraud measures analogous to those discouraging fee-for-service “upcoding.”

## Conclusions

Metrics matter. Valuation and remuneration of health added would improve health system efficiency and lead to greater population health. The methods to compute a health-added metric now exist. It remains to be seen whether this technical ability will be animated by market traction, regulatory impetus, or visionary leadership.
